# Adherence assessment in post-cardiac arrest care: are physiological targets sufficient? Author

**DOI:** 10.1186/s13613-025-01559-4

**Published:** 2025-09-25

**Authors:** George Mannu

**Affiliations:** Emergency Medicine, Prince Charles Hospital, Gurnos Rd , Merthyr Tydfil , Wales CF47 9DT UK

Letter to the Editor,

I read with interest the recent study by Merigo et al. on adherence to post-cardiac arrest care guidelines and its relationship with survival and neurological outcomes [1]. The team deserves recognition for carrying out such a detailed evaluation, especially in a setting where standardising care is inherently challenging.

The improvement in adherence to guideline recommendations, particularly in haemodynamic management, is reassuring. Nonetheless, it is notable that these gains did not translate into statistically significant changes in survival or neurological outcomes. This raises several important questions.

To begin with, I wonder whether the way adherence was defined, based mostly on physiological measures rather than clinician intent, may have limited the ability to fully capture the effects of clinical decisions. For example, were patients considered nonadherent even when appropriate treatments were given but targets were not met due to refractory shock?

Ventilation adherence was also consistently low. While the authors note that most deviations were minor, could even small changes in PaO2 or PaCO2 influence outcomes in select patients? Did the authors perform any subgroup analysis to explore this further?

Another point worth exploring is the rise in survival seen in the most recent cohort. Could this improvement be partly due to broader developments in critical care rather than the guidelines themselves? It would be helpful to know whether adjusting for secular trends or changes in ICU practice affected this apparent improvement over time.

Lastly, I am curious how guideline implementation changed across the study period. Were there structured efforts, such as staff education or protocol updates, that facilitated improved adherence?

This work contributes meaningful data to a key area, but it also highlights the need to better understand how guideline-based care influences long-term outcomes. I look forward to future studies that build on these findings, ideally across multiple centres.

## Data Availability

Not applicable.

## Abbreviations

ICU: intensive care unit
PaO2: partial pressure of arterial oxygen
PaCO2: partial pressure of arterial carbon dioxide
